# Stress-Recovery State in Fibromyalgia Patients and Healthy People. Relationship with the Cardiovascular Response to Stress in Laboratory Conditions

**DOI:** 10.3390/ijerph17093138

**Published:** 2020-04-30

**Authors:** Borja Matías Pompa, Almudena López López, Miriam Alonso Fernández, Estefanía Vargas Moreno, José Luis González Gutiérrez

**Affiliations:** Psychology Department, Rey Juan Carlos University, 28922 Alcorcón, Spain; almudena.lopez@urjc.es (A.L.L.); miriam.alonso@urjc.es (M.A.F.); estefania.vargas@urjc.es (E.V.M.); joseluis.gonzalez@urjc.es (J.L.G.G.)

**Keywords:** fibromyalgia, stress-recovery state, sympathetic nervous system, stress, cardiovascular response

## Abstract

The current study´s objective was to determine the relationship between stress-recovery state and cardiovascular response to an acute stressor in a sample of female fibromyalgia patients in comparison with a control group of healthy participants. The laboratory procedure was completed by 36 participants with fibromyalgia and by 38 healthy women who were exposed to an arithmetic task with harassment while blood pressure and heart rate were measured during task exposure.

## 1. Introduction

In recent decades, stress-recovery processes (e.g., sleep, motivated behavior like eating and drinking, or goal-oriented components like relaxation and meeting friends) have gained prominence as mechanisms necessary to the proper functioning of the stress response system [1,2]. Various studies have shown them to be linked to positive outcomes; for example, resilience or vigor and with a minor presence of negative conditions such as depression, anxiety, anger, fatigue, negative affect or confusion [2]. Overall, the presence of a good stress-recovery state (a balance between exposure to sources of stress and the benefit of major sources of recovery) has been noted as a prerequisite to coping well with stressors from a physiological point of view [1,3]. In this sense, dysfunctional physiological responses against stressors are well-known predictors of poor health. Specifically, this is the case with an excessive or insufficient cardiovascular response against stress, which has been shown to be associated with poorer perceived health [4] and a greater presence of pain [5,6]. 

On the basis of the relationship described above between healthy people´s prevalent stress-recovery state and their capacity to respond to acute stressors, Kallus and Kellmann have pointed out the importance of recovery in everyday life [1,3]. However, little is known about the role of the stress-recovery state when chronic dysregulation of the physiological stress response occurs. This would be the case of pathologies such as irritable bowel, chronic fatigue syndrome, migraine headaches, depression, posttraumatic stress disorder or fibromyalgia [7]. In such circumstances, partial or complete autonomy of the physiological stress response may be hypothesized as stress dysregulation and involves changes in the ability to respond to the inputs that persist on an ongoing basis. Consequently, it is expected that a partial or complete disconnection between the individual stress-recovery state and the physiological response to stress may be observed in sufferers of these disorders, compared to healthy people.

Fibromyalgia (FM) is a chronic disease characterized by fatigue and widespread pain in the muscles and connective tissues. It is seen as a stress-related disorder characterized by the presence of adverse life events, such as emotional, physical and sexual abuse in childhood and adulthood, as well as by a life history of chronic stress [8,9]. It has been observed that the physiological response to stress is altered in FM, basically resulting in a fundamental and persistent loss of adaptability as a result of the exhaustion of the system [5]. This alteration has been presented as a shift from a state of hyperfunction to one of hypofunction, leading finally to an inability to respond to future mental and physical stressors. Thus, reduced cortisol levels and increased autonomic activity have been observed at basal periods, in parallel with a blunted autonomic activity (hyporeactivity) under stress conditions. As a chronic response extended over time, this hyporeactivity may be associated with a global functional independence from daily sources of stress and recovery. In other words, it would be expected that both stress and recovery sources may no longer be able to influence the physiological response to acute stressors of these patients. However, in a study by Bojner-Horwitz et al. [10], a blunted circadian cortisol rhythm in fibromyalgia patients was restored to normal after a period of dance therapy (a form of recovery process), indicating that the link between the functioning of the stress response and the exposure to recovery processes may be maintained. If this were the case, promoting recovery processes could mean an improvement in the life quality of these people, as it is already the case in healthy people. In consequence, the lines of treatment to be followed with fibromyalgia patients could be different and even divergent, which would lead to a rethinking of the therapeutic approach to stress management for these patients.

Thus, the present study examines the possible differences existing in the relationship between stress and recovery processes and cardiovascular response to a laboratory-induced, trauma-unrelated stressor in a sample of female FM patients and a control group of healthy participants.

## 2. Materials and Methods 

### 2.1. Participants

The laboratory procedure was completed by 36 participants diagnosed with FM, according to the American College of Rheumatology criteria [11], and by 38 healthy women. All participants were Caucasian and lived in the Community of Madrid. In the healthy group, most participants had completed secondary or higher education (84%) and the most frequent marital status was married (84.2%). The FM group had a similar profile, with most people having completed secondary or higher education (73%) and being married (72%). The inclusion criteria for both groups were: aged between 25 and 65, normal or corrected-to-normal vision, and the ability to read and write in Spanish to eighth-grade equivalent level. General exclusion criteria were: Body Mass Index higher than 30 (increased risk of cardiovascular disease), significant acute or chronic medical or psychiatric disorders (aside from FM in the FM sample), current regimens of psychoactive medication (except low-dose benzodiazepines—10 mg of diazepam per day or equivalent—and tricyclic antidepressants—50 mg of amitriptyline per day or equivalent) to reduce the risk of potential effects on arterial blood pressure and heart rate [12], and incompatible life circumstances such as night working, lactation or recent surgery. An additional specific exclusion criterion for patients with FM was the presence of other pain disorders previous to FM diagnosis. Participants were also excluded from the healthy control group when a self-reported history of chronic pain was presented, or pain was reported at the time of the study.

In our case, beyond the final sample of 74 participants, 26 potential participants were excluded from the group of healthy controls on the grounds of a self-reported history of chronic pain (epicondylitis, disc herniation or similar). Three potential patients were excluded from the FM group, one of them as a consequence of using fentanyl patches, and the other two due to high doses of tricyclic antidepressants.

The sample size was sufficient to detect a medium effect size (δ = 0.15) in moderation analysis with a target power of 0.80, which is adequate following Cohen’s guidelines for small, medium and large effects [13].

The mean age of FM participants was 53.81 years (SD = 8.23), and time since first pain symptoms was 18.81 years (SD = 13.47). Pain intensity mean was 6.45 (SD = 8.23) on a scale of 0 (“no pain”) to 10 (“pain as bad as you can imagine”). The mean age of the healthy controls subsample was 48.66 years (SD = 8.42). Women diagnosed with fibromyalgia were recruited from the Association of Patients with Fibromyalgia and Chronic Fatigue Syndrome of the Community of Madrid (AFINSYFACRO) and from the pain management unit at Hospital Foundation of Alcorcón. Healthy controls were recruited among the parents of students at a Madrid university. 

All subjects gave their informed consent for inclusion before they participated in the study. The study was conducted in accordance with the Declaration of Helsinki, and the protocol was approved by the Ethics Committee of researcher´s work center (PSI2010-21888).

### 2.2. Questionnaire Measures

Pain intensity was measured by calculating the average of four numerical rating scales from 0 (“no pain”) to 10 (“pain as bad as you can imagine”) for the conditions of “worst pain”, “least pain” and “average pain”, as well as for “current pain”. These scales are included in the Brief Pain Inventory, which has been recommended as a core pain measure by the Initiative on Methods, Measurement, and Pain Assessment in Clinical Trials (IMMPACT) [14]. 

Participants’ past and present stress experience was evaluated by a set of instruments. First, severity of childhood abuse and neglect was evaluated with the Childhood Trauma Questionnaire-short form (CTQ-SF), which was created to address the need for reliable and valid assessment of a broad range of maltreatment [15]. Second, total presence of potentially traumatizing experiences and their severity were assessed by means of the Traumatic Experiences Checklist (TEC) [16]. Third, daily hassles and daily uplifts were measured with the Hassles and Uplifts Scale (HSUP) [17]. This measure includes evaluations of positive and negative events occurring in each person’s daily life defined as “hassles” and “uplifts” (e.g., meeting deadlines or goals at work, enough money for necessities, taking care of paperwork, etc.) Finally, the frequency of significant life changes (e.g., marriage, confinement in jail or comparable institution, death of spouse, etc.) and their negative and positive impacts on the participants were evaluated using the Life Experiences Survey (LES) [18].

Participants were asked to rate their level of emotional arousal during the laboratory session using three unipolar visual analogue scales (VAS) for anxiety, sadness and anger, with two anchors labeled “no anxiety/sadness/anger” and “severe anxiety/sadness/anger”. The validity of VAS for measuring emotional states has been widely recognized for some time [19,20].

Expectations about how stressful the participants would find the task were assessed immediately before attempting it using a 7-point Likert scale with 1 representing “not stressing” and 7 representing “very stressing”. At the same time, beliefs about their perceived ability to successfully tackle the task were assessed using a 7-point Likert scale with 1 representing “not competent at all” and 7 representing “competent”. Measuring efficacy expectations by means of single-item, Likert-type responses has been used successfully in previous research [21]. 

Finally, the stress-recovery state was evaluated with the Recovery-Stress Questionnaire (REST-Q) [22]. It evaluates different stress-recovery state factors grouped in twelve scales that give information about both demanding conditions, namely “sources of stress” or “stress processes” (general, social and emotional stress, conflicts, fatigue, lack of energy and physical complaints) and rest or recovery activities performed during the last three to four days, namely “sources of recovery” or “recovery processes” (success, social recovery, physical recovery, general well-being and sleep quality). These scales can be grouped into two basic factors: “General Stress” (which captures most of the variance in the level of exposure to sources and processes of stress) and “General Recovery” (which captures most of the variance in the level of exposure to sources and processes of recovery). The general stress factor covers two subfactors in turn, named “general/emotional stress” and “performance-related/work-related stress”. The questionnaire is composed of 48 items whose frequency is rated on a 7-point scale: 0 (never), 1 (seldom), 2 (sometimes), 3 (often), 4 (more often), 5 (very often) and 6 (always). Some examples of items for the three main factors are: General recovery (e.g., “I visited some close friends”, “I had a satisfying sleep”, “I felt as if I could get everything done”, “I made important decisions”), General/emotional stress (e.g., “I was fed up with everything”, “I felt anxious or inhibited”, “I was angry with someone”, “I was in a bad mood”) and Performance-related/work-related stress (e.g., “I worried about unresolved problems”, “I was tired from work”, “I had difficulties in concentrating”, “I felt physically exhausted”). The REST-Q possesses adequate reliability and validity [1,23]. 

Specifically, from a theoretical point of view, REST-Q is based on a biopsychosocial approach to stress. It integrates the classic Lazarus and Folkman’s transactional model of stress [24] with a new action-oriented approach where availability of resources is pivotal. In this way, Kallus and Kellmann [25] highlight the role of recovery, which should not be understood as a passive process, but as an active and independent phenomenon that allows resources to be continuously available to cope with stressors. Thus, recovery is not only an active ingredient of stress perception. Instead, it also refers to the availability of those resources that will influence coping from a physiological and psychological point of view.

### 2.3. Body Mass Index 

Body mass index (BMI) was calculated as the ratio of participants’ height to weight using the formula BMI = (weight in kilograms)/(height in meters)^2^. 

### 2.4. Cardiovascular Recording

Systolic blood pressure (SBP), diastolic blood pressure (SDP) and heart rate (HR) were measured using beat-to-beat digital plethysmography (Finometer^®^, Finapres Medical Systems BV (FMS), Amsterdam, The Netherlands). The inflatable blood pressure cuff was placed on the third finger of the nondominant hand. The Finometer computed all cardiovascular variables using Beatscope Easy^®^. Finometer has been shown to track intra-arterial readings extremely well, even during sudden changes of blood pressure (BP) and heart rate (HR) [26], making it a useful tool for cardiovascular reactivity and recovery testing. In addition, as a beat-to-beat technique it is extremely reliable because of the large number of blood pressure and heart rate measurements that are averaged [27].

### 2.5. Stress Task: Mental Arithmetic with Harassment

Participants were asked to count backwards by thirteen as quickly and accurately as possible, starting from 2036. The specific instruction was “The task you are going to do next is to count backwards by thirteen from the number 2036. You have to do it as quickly and accurately as possible. I’ll tell you through the intercom when you can start”. While the participants counted backwards, they were harassed and interrupted repeatedly by the experimenter. The timing and content of these interruptions were standardized and independent of the participant’s performance. Specifically, there were three interruptions at 30 s, 90 s and 120 s. In the first one, the comment was, “You are going to have to start again but, this time, you will count by sevens to make it easier for you”, the second one was, “You are going to start counting again from 2036 by sevens because you’re making some mistakes that don’t allow us to continue”, and the last one was, “You are going to repeat it one more time and, if you do not speed up, we are not going to be able to use your data”. Negative verbal harassment of this sort has shown a high capacity to generate cardiovascular response due to the emotional component associated with the implicit feeling of uncontrollability and social evaluation [28,29,30]. The stress task lasted three minutes. 

### 2.6. Procedure

The laboratory sessions took place between 10:00 and 14:00. Once the written informed consent, self-assessment questionnaire and affect ratings were provided (including REST-Q), participants were fitted with a finger blood pressure cuff while seated in a comfortable armchair. They were then asked to remain quiet during a 12-min baseline rest period with the experimenter out of the room, which is sufficient time to ensure adequate stability for the measurement [31]. Shortly after this baseline period, the experimenter gave the instructions for the mental arithmetic task and asked participants to report their expectations regarding the stress potential of the arithmetic task and their competence to accomplish it. The experimenter then left the room and when the participants finished the task (3 min), returned to take off the finger blood pressure cuff and ask them to complete the affect questionnaire again.

For the statistical analyses, baseline values were computed as the mean of individual systolic blood pressure, diastolic blood pressure and heart rate measurements taken during the final five minutes of the initial rest period. Reactivity values were computed as the mean of the individual measurements taken during the three minutes of the arithmetic task performance minus the mean of baseline values.

### 2.7. Statistical Analysis

#### 2.7.1. Preliminary Analyses

Data were analyzed using SPSS 22 Statistical Software (IBM, Chicago, IL, USA). All reported results were considered to be significant at the *p* ≤ 0.05 level and were considered a trend toward significance at *p* ≤ 0.10. All data were tested for the presence of outliers prior to analysis (no outliers were detected), and tested for normality using Kolmogorov–Smirnov and Shapiro–Wilks tests. As some variables had non-normal distributions, the non-parametric Mann–Whitney U test was used systematically to examine for potential differences among both groups in sociodemographic and clinical characteristics (age and pain intensity). When differences emerged in any of these characteristics, transformations were made as required to allow inclusion as covariates in all remaining models and adjust for these differences. 

Finally, to establish the self-reported stress levels of the sample and the potential differences among both groups in the three REST-Q factors (General/emotional stress, Performance-related/work-related stress, and General recovery), the non-parametric Mann–Whitney U test was again used.

#### 2.7.2. Cardiovascular Responses to the Stressor Task

In order to control potentially relevant variables, and because the sample was sufficient to use the F test, which is robust against possible Type 1 and Type 2 errors, ANCOVA and mixed ANOVA were carried out for the analyses of this section.

Mixed ANOVA was employed to ascertain the effectiveness of the arithmetic task in eliciting mood change (sadness, anxiety and anger) and cardiovascular response change (SBP, DBP and HR), as well as to examine for potential differences between groups at baseline and in the possible changes caused by the task.

ANCOVA was used to test for potential differences in the participants’ expectations of how stressful they would find the task, as well as in their perceived ability to successfully tackle the task before proceeding.

#### 2.7.3. Influence of Stress-Recovery Processes on the Cardiovascular Response

With the goal of examining how stress-recovery processes influenced the cardiovascular response to an acute stressor in both groups, moderation analyses with the macro PROCESS of SPSS were performed [32]. This macro was used because it employs a bootstrapping method which does not require the variables to fit a normal distribution. 

The three factors of the REST-Q questionnaire (General/emotional stress, Performance-related/work-related stress, and General recovery) were used specifically as individual predictor variables and the group as a moderator variable (FM group and control group). Moderation analyses were performed for systolic pressure, diastolic pressure and heart rate at the baseline level and the response to the stressor task (reactivity). As specified in the preceding section, the previously specified potential covariates were controlled if necessary. 

Prior to the analysis, change scores for reactivity levels were computed using the difference between the mean of the task period and the mean of the baseline period. Raw change scores rather than residualized change scores were used, as recommended by Llabre et al. [33]. In addition, this method allowed us to control for possible influences of the baseline variables in the reactivity moderations. 

## 3. Results

### 3.1. Preliminary Analyses 

Non-parametric comparisons to examine for possible differences in the baseline of both groups showed that significant differences in marital status, BMI, education level and number of children in their care did not exist. However, the average age of the FM group was significantly higher than that of the control group (U = 433.500; *p* = 0.007). For this reason, a square-root transformation of this variable was performed to obtain normality. 

As expected, the FM group scored significantly higher than the control group in pain intensity, with averages on a scale from 0 to 5 of 2.22 (SD = 1.35) and 0.23 (SD = 0.49), respectively (U = 83; *p* = 0.000).

As expected, the two groups had significantly different scores on most of the scales to measure stress, as can be seen in Table 1, except in positive impact of life changes (LES positive change score). Comparisons between groups showed that the FM group scored higher than the control group on severity of childhood abuse and neglect (CTQ), total presence of potentially traumatizing experiences (TEC total presence score), total severity of trauma (TEC total severity score), daily hassles (HSUP), occurrence of significant life changes (LES frequency) and negative impact of life changes (LES negative change score). The FM group scored lower than the control group on daily uplifts (HSUP). 

**Figure 1:**
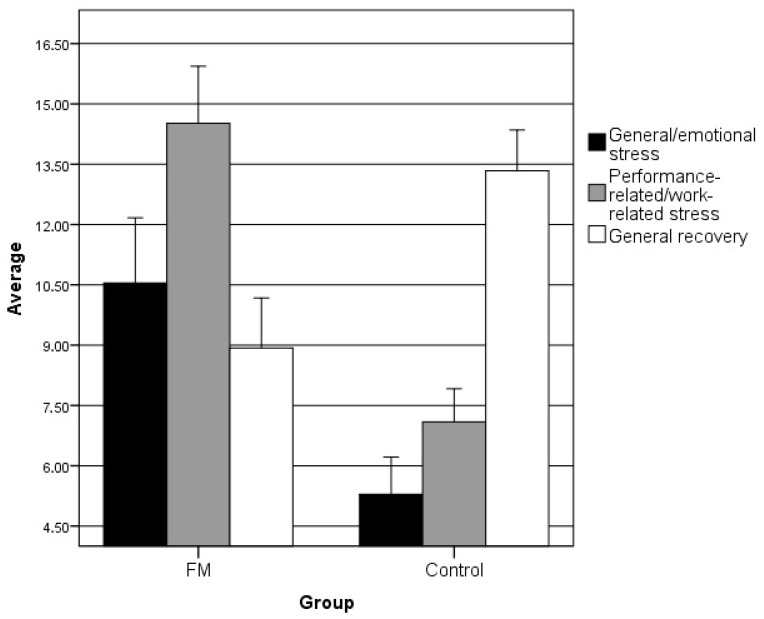
Average score of the groups in each of the REST-Q factors. As expected, stress levels were higher in the FM group and recovery levels were higher in the healthy group.

In the REST-Q factors, the FM group showed significantly higher scores than the control group in the factors of “General/emotional stress” (U = 213.500; *p* < 0.001) and “Performance-related/work-related stress” (U = 101.500; *p* < 0.001), but lower scores in “General Recovery” (U = 225; *p* < 0.001), as can be seen in Figure 1. 

### 3.2. Cardiovascular Responses to the Stressor Task

In the following analyses, only age was included as a covariate according to the preliminary analyses performed.

Regarding the mood variables, the mixed ANOVA showed a main time effect (baseline-task) for the following moods: anxiety (F (1,69) = 203.420; *p* < 0.001; η2 = 0.747), sadness (F (1,69) = 21.209; *p* < 0.001; η2 = 0.235), and anger (F (1,69) = 42.127; *p* < 0.001; η2 = 0.379). Specifically, participants reported being sadder, angrier and more anxious during the speech task compared to baseline. The interaction effect between time and group was not significant for any of mood variables, so no significant differences were observed between the groups in the three measures of mood change: anxiety (F (1,69) = 3.508; *p* = 0.065; η2 = 0.048), sadness (F (1,69) = 0.317; *p* = 0.576; η2 = 0.005), and anger (F (1,69) = 0.075; *p* = 0.785; η2 = 0.001). However, a certain tendency close to significance was observed in anxiety, with the highest values belonging to the control group.

At the cardiovascular level, the mixed ANOVA revealed that the individual main time effect (baseline-task) was significant on systolic blood pressure (F (1,72) = 167.045; *p* < 0.001; η2 = 0.699), diastolic blood pressure (F (1,72) = 213.828; *p* < 0.001; η2 = 0.748) and heart rate (F (1,72) = 210.843; *p* < 0.001; η2 = 0.745). Specifically, participants reported higher scores in all three during the task than at baseline.

Regarding cardiovascular baseline scores, no significant differences were founded in any variable examined between the groups: SBP (F (1,72) = 0.287; *p* = 0.594; η2 = 0.004); DBP (F (1,72) = 1.364; *p* = 0.247; η2 = 0.019); HR (F (1,72) = 2.062, *p* = 0.155; η2 = 0.03). In relation to the interaction between time and group, a significant effect on both systolic blood pressure F (1,72) = 6.889; *p* = 0.011; η2 = 0.087 and heart rate (F (1,72) = 14.704, *p* = 0.000; η2 = 0.170) was observed. Therefore, significant differences were observed between the groups in both measurements, with a higher change for the control group scores.

In relation to their expectations regarding how stressful they would find the task F (1,72) = 0.743; *p* = 0.392; η2 = 0.010, as well as their perceived ability to tackle the task successfully F (1,72) = 0.020; *p* = 0.887; η2 = 0.000, the ANCOVA test did not show significant differences between the groups in either of the two variables.

### 3.3. The Group as a Moderating Factor in the Relationship between Stress-Recovery Processes and Cardiovascular Response

Different moderation analyses were performed for baseline and reactivity measurements, as can be seen in Table 2 and Table 3, respectively. This was done for the purpose of seeing if the stress-recovery state had an influence only in the cardiovascular reactivity period or also in the cardiovascular baseline period. It should be noted that given the previous analyses described, it was only appropriate to include age as a covariate for the analysis of the group’s moderating role on the relationship between Performance-related/work-related stress factor and systolic blood pressure. This made it possible to appreciate that the baseline measurements had no moderating effects. However, for reactivity measurements, three moderating effects of the group were identified, two of them for the relationship with the Performance-related/work-related stress factor (Beta = 1.78 for SBP and Beta = 1.10 for DBP) and the other for the relationship with the General recovery factor (Beta = 0.94 for HR).

**Figure 2:**
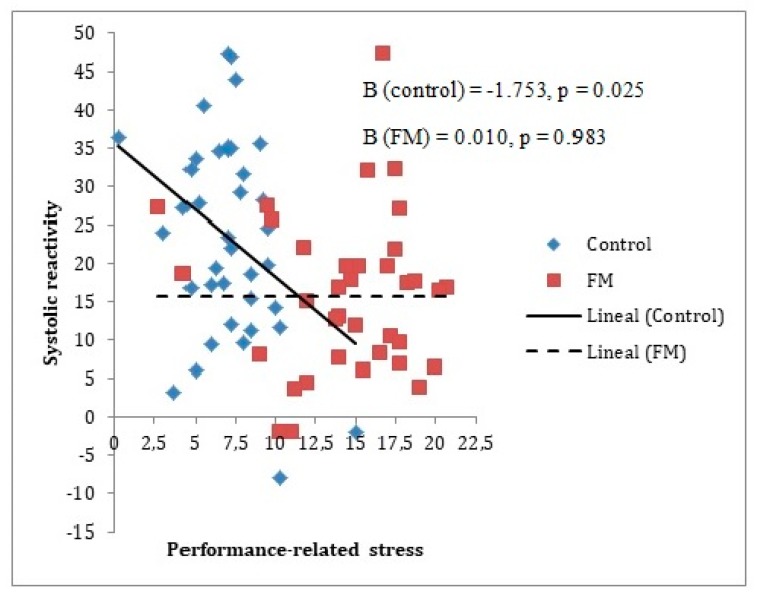
Group moderating role between a systolic reactivity measure and Performance-related stress factor. The reactivity index is represented as the difference between the baseline and the stress measurements (task minus baseline). In the figure, the lines represent raw scores. As can be observed, only in the control group does systolic pressure reactivity decrease significantly when Performance-related stress factor levels increase. Note: FM = fibromyalgia; Systolic reactivity is measured in mmHg = millimeters of mercury.

**Figure 3:**
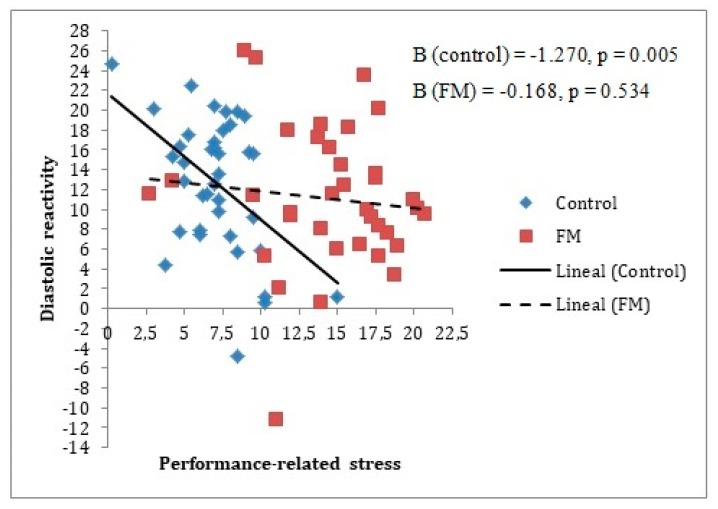
Group moderating role between diastolic reactivity measure and Performance-related stress factor. The reactivity index is represented as the difference between the baseline and the stress measurements (task minus baseline). In the figure, the lines represent raw scores. As can be observed, only in the control group does diastolic pressure reactivity decrease significantly when Performance-related stress factor levels increase. Note: FM = fibromyalgia; Diastolic reactivity is measured in mmHg = millimeters of mercury.

In Figure 2 and Figure 3, it can be observed that an increase in the Performance-related/work-related stress factor levels was associated with a decrease in systolic reactivity and diastolic reactivity; this was only the case for the control group, with no relationship between both variables in the FM group.

**Figure 4:**
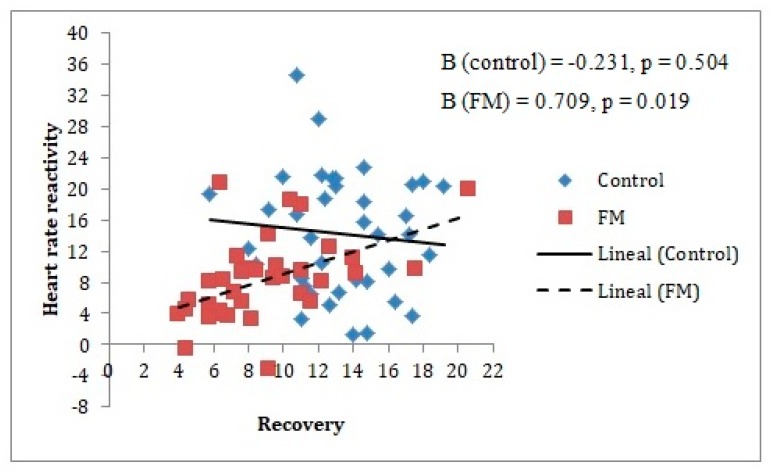
Group moderating role between heart rate reactivity measure and General recovery factor. The reactivity index is represented as the difference between the baseline and the stress measurements (task minus baseline). In the figure, the lines represent raw scores. As can be observed, only in the FM group does heart rate reactivity increase significantly when General recovery factor levels increase. Note: FM = fibromyalgia; Heart rate reactivity is measured in bpm = beats per minute.

In the case of the General recovery factor, it was noted how an increase in this factor was related to an increase of heart rate reactivity in the stress task, but only in the FM group. This was not observed in the control group, as can be seen in Figure 4. 

## 4. Discussion

The aim of the current study was to examine how the stress-recovery state (the level of exposure to sources of stress and sources of recovery) was related to the cardiovascular response of a group of female patients with FM, in comparison with a group of healthy women. Both groups were exposed to a mental arithmetic task with harassment, during which their cardiovascular response was measured through systolic blood pressure, diastolic blood pressure and heart rate. 

As expected, the preliminary analyses showed that the FM group had greater impact and frequency of traumatic experiences, life events and daily situations than the control group, as well as a higher number of stress sources in the previous three days, while the control group had a higher number of recovery sources in the same period. In addition, although there was no evidence of a predominant baseline response in the FM group in comparison with the control group in the cardiovascular analyses, the FM group was observed to have a blunted cardiovascular response to the stress task in systolic blood pressure and heart rate. This result is consistent with the scientific evidence that supports the existence of chronic dysregulation of the response system to stress in people with FM [5].

Regarding the relationship between stress-recovery state and cardiovascular response, no moderation effect by group was observed at baseline for either stress or recovery. However, in the reactivity analyses, something different occurred. A greater presence of stress sources during the three days prior to the laboratory stress task was associated in the control group with a decrease in systolic and diastolic reactivity in laboratory conditions. This circumstance has usually been linked in previous research to potentially suffering from diverse types of diseases [4]. In contrast, the cardiovascular response in the FM group was not linked to a greater or lesser presence of stress during the previous three days.

Regarding the relationship between sources of recovery and cardiovascular reactivity, it was shown that a greater presence of recovery sources was related to a stronger cardiovascular response in the FM group. This result is consistent with previous scientific data showing that a blunted circadian cortisol rhythm in fibromyalgia patients can be restored to normal after a period of dance therapy [10]. Thus, recovery sources seem to work as significant resources for resistance to stressors, which may have significant implications [3,25]. First, beyond the presence or absence of stressors, access to recovery sources may constitute a crucial factor in gaining cardiovascular management of acute stressors by FM sufferers. Second, against the possibility of a complete decoupling between the stress-recovery state and the physiological response to stress of FM patients, a partial dependency between them was still observed, which opens the door to studying recovery sources as a way of regulating the physiological alterations in the stress response of these patients, as evidenced by Bojner-Horwitz, Theorell, and Anderberg [10]. Future research should examine whether this circumstance may be generalizable to other pathologies characterized by a chronic dysregulation in the stress system. 

Some limitations of the present study should be mentioned. First, this study has sufficient power to detect medium-sized differences between groups with regard to their cardiovascular response. These results show the significant role of stress-recovery state in the cardiovascular response to stressors of patients with FM. Nevertheless, a greater sample size would have been desirable in order to observe even small differences. Second, cardiovascular responses do not provide a direct measure of sympathetic–adrenal–medullary activation as they are influenced by inputs from multiple systems [34]. This may partially explain the minor discrepancies observed in heart rate and blood responses. Employment of more direct measures of the sympathetic function (e.g., catecholamines) may benefit future studies. Third, despite the fact that the present study has yielded homogeneous evidence with women, such results are not a priori generalizable to men. Diverse psychosocial and biological factors have been proposed to explain the higher prevalence of FM among women, some of them (e.g., hormone related) potentially on the basis of the cardiovascular responses to stressors [35]. Consequently, future studies could replicate this study in men with FM by paying special attention to possible differences in their cardiovascular responses. Fourth, since the cardiovascular effects of therapeutic doses of benzodiazepines are only observable in older patients (e.g., ≥ 60 years old) [36], and therapeutic doses of tricyclic antidepressants are correlated with minor increases in systolic and diastolic blood pressure and heart rate [37], it is not expected that the very low therapeutic doses allowed in the present study significantly altered the observed cardiovascular responses. Nevertheless, given the lack of data regarding potential interactions between the use of these substances and the presence of FM in terms of cardiovascular responses, it seems necessary to consider this circumstance in the interpretation of the present results. Fifth, since it is a cross-sectional design, years of illness are expected to influence the ability to access and use sources of recovery. In this sense, it is unknown how this circumstance may affect the impact of recovery on the reactivity to stress of these patients. For future studies, it could be interesting to use a longitudinal design, with the aim of examining the development of the cardiovascular response to acute stressors by controlling progressive changes in the stress-recovery state. Finally, although mental arithmetic with harassment is assumed to possess some of the elements that define real-life stressors (time and cognitive pressure in combination with frustration and threat to self-esteem) and its use has been the norm in comparable studies, the use of more ecological assessments has been called for [38].

## 5. Conclusions

In conclusion, the current study indicates that sources of stress and recovery may act differently on individuals’ cardiovascular functioning in response to stress depending on whether they are in an FM or a healthy group. Thus, it was the presence of stress sources in the control group that was related to a decrease in cardiovascular reactivity to acute stressors, while in the FM group, it was the recovery sources that were related to an increase in cardiovascular response to stressors, not the stress sources. Given the need for an adequate and sufficient cardiovascular response in mobilizing the energy to activate the necessary mechanism for managing stressors [39], a hypoactive cardiovascular response may increase the likelihood of suffering other kinds of diseases [4]. In addition, a hypoactive response could be a significant factor in vulnerability to subsequent stress and pain [40,41]. 

Both in the case of the FM group and in the healthy control group, the present study points to a relationship between a healthy stress-recovery state and an adequate and sufficient cardiovascular response. Specifically, it provides preliminary evidence of the importance of recovery sources and their relationship with the response to stress in FM patients. This opens the door to implementing research lines aimed at analyzing the therapeutic strategy of fostering recovery as a route to improving the cardiovascular response of patients in acute stress situations. In this sense, together with treatment strategies to reduce stress which are regularly applied in this population (e.g., relaxation, revaluation and managing stressors) [42,43], the efficacy of strategies based on the increase of recovery processes, such as social and leisure activities, rest, or the improvement of sleep quality should be examined. These vital areas are currently an object of intervention for decreasing symptoms and increasing the quality of life in FM patients [44]. However, it is not known whether they have a functional capacity to normalize the cardiovascular response to stressors. In the affirmative case, such training should play a central role in the treatment plan, based on previously modifying overexertion of cognitive schemata and appropriate work on goal selection [45].

## Figures and Tables

**Table 1:** U Mann-Whitney Test results for the significant differences between groups in the frequency and impact of traumatic experiences, life events and daily situations.

	Fibromyalgia			Controls			U Mann-Whitney
	Mean	SD	Median	Mean	SD	Median	
Severity of Childhood Abuse and Neglect (CTQ)	49.95	17.24	46	41.21	11.51	38.5	440 **
Potentially Traumatizing Experiences (TEC)							
Total Presence	7.03	4.36	7	3.76	2.93	3	358.500 ***
Total Severity	3.78	3.24	3	1.74	1.84	1	382.500 **
Hassles and Uplifts (HSUP)							
Hassles	40.33	21.99	40.5	28.66	17.09	25	460 *
Uplifts	45.61	19.57	45	60.45	26.44	60	469 *
Occurrence of Significant Life Changes (LES)							
Frequency	6.94	3.63	6.5	3.84	3.61	3	365.500 **
Negative Change	−10.05	7.01	−11	−4.26	4.06	−3	346.500 ***
Positive Change	3.89	4.06	3	2.82	3.95	2	539

* *p* < 0.05; ** *p* < 0.01; *** *p* < 0.001.

**Table 2:** Moderation analyses results for the prediction of SBP, DBP and HR at baseline.

			SBP ^1^					DBP ^2^					HR ^3^		
	Beta	SE	T(p)	Inc.R^2^	F(p)	Beta	SE	T(p)	Inc.R^2^	F(p)	Beta	SE	T(p)	Inc.R^2^	F(p)
General Stress	−0.24	0.55	−0.44(0.66)	-	-	0.26	0.38	0.69(0.49)	-	-	−0.13	0.37	−0.36(0.72)	-	-
Group	−9.21	8.73	−1.05(0.29)	-	-	1.28	6.02	0.21(0.83)	-	-	−6.79	5.82	−1.17(0.25)	-	-
General Stress × Group	0.74	1.12	0.66(0.51)	0.01	0.44(0.51)	−0.72	0.77	−0.94(0.35)	0.01	0.88(0.35)	0.49	0.74	0.67(0.51)	0.01	0.45(0.51)
Performance-Related Stress	0.57	0.63	0.91(0.36)	-	-	0.46	0.43	1.09(0.28)	-	-	0.28	0.41	0.70(0.49)	-	-
Group	3.76	12.81	0.29(0.77)	-	-	8.55	8.70	0.98(0.33)	-	-	−16.1	8.35	−1.93(0.06)	-	-
Performance-Related Stress × Group	−1.19	1.25	−1.00(0.34)	0.01	0.91(0.34)	−1.39	0.86	−1.62(0.11)	0.04	2.64(0.11)	0.97	0.82	1.18(0.24)	0.02	1.38(0.24)
General Recovery	0.31	0.57	0.55(0.58)	-	-	0.14	0.39	0.35(0.73)	-	-	−0.54	0.38	−1.42(0.16)	-	-
Group	−11.9	14.01	−0.85(0.40)	-	-	−10.5	9.57	−1.10(0.27)	-	-	−5.43	9.14	−0.59(0.55)	-	-
General Recovery × Group	0.76	1.14	0.67(0.51)	0.01	0.44(0.51)	0.73	0.79	0.92(0.36)	0.01	0.86(0.36)	−0.05	0.75	−0.06(0.95)	0.00	0.00(0.95)

Note: ^1^ systolic blood pressure; ^2^ diastolic blood pressure; ^3^ heart rate.

**Table 3:** Moderation analyses results for the prediction of SBP, DBP and HR at reactivity.

			SBP ^1^					DBP ^2^					HR ^3^		
	Beta	SE	T(p)	Inc.R^2^	F(p)	Beta	SE	T(p)	Inc.R^2^	F(p)	Beta	SE	T(p)	Inc.R^2^	F(p)
General Stress	−0.74	0.41	−1.83(0.07)	-	-	−0.37	0.23	−1.57(0.12)	-	-	−0.16	0.23	−0.69(0.49)	-	-
Group	−10.2	6.42	−1.58(0.12)	-	-	−3.83	3.68	−1.04(0.30)	-	-	−6.68	3.60	−1.85(0.07)	-	-
General Stress × Group	0.81	0.81	1.00(0.32)	0.01	1.00(0.32)	0.49	0.47	1.05(0.30)	0.01	1.10(0.30)	0.15	0.45	0.32(0.75)	0.00	0.11(0.75)
Performance-Related Stress	−0.88	0.45	−1.93(0.06)	-	-	−0.72	0.26	−2.79(0.01)	-	-	−0.55	0.25	−2.20(0.03)	-	-
Group	−20.3	9.24	−2.20(0.03)	-	-	−8.06	5.24	−1.54(0.13)	-	-	−11.9	5.18	−2.30(0.02)	-	-
Performance-Related Stress × Group	1.78	0.91	1.95(0.05)	0.05	3.82(0.05)	1.10	0.52	2.14(0.04)	0.06	4.56(0.04)	0.89	0.50	1.78(0.08)	0.03	3.16(0.08)
General Recovery	0.30	0.42	0.72(0.48)	-	-	0.13	0.24	0.55(0.58)	-	-	0.19	0.23	0.82(0.42)	-	-
Group	−19.6	10.19	−1.93(0.06)	-	-	−6.69	5.94	−1.13(0.26)	-	-	−15.9	5.50	−2.89(0.01)	-	-
General Recovery × Group	1.20	0.84	1.43(0.16)	0.03	2.04(0.16)	0.48	0.49	0.98(0.33)	0.01	0.58(0.33)	0.94	0.45	2.07(0.04)	0.05	4.29(0.04)

Note: ^1^ systolic blood pressure; ^2^ diastolic blood pressure; ^3^ heart rate.
